# Exploring cell-derived extracellular vesicles in peripheral blood and bone marrow of B-cell acute lymphoblastic leukemia pediatric patients: proof-of-concept study

**DOI:** 10.3389/fimmu.2024.1421036

**Published:** 2024-08-21

**Authors:** Fábio Magalhães-Gama, Marina Malheiros Araújo Silvestrini, Juliana Costa Ferreira Neves, Nilberto Dias Araújo, Fabíola Silva Alves-Hanna, Marlon Wendell Athaydes Kerr, Maria Perpétuo Socorro Sampaio Carvalho, Andréa Monteiro Tarragô, Gemilson Soares Pontes, Olindo Assis Martins-Filho, Adriana Malheiro, Andréa Teixeira-Carvalho, Allyson Guimarães Costa

**Affiliations:** ^1^ Programa de Pós-graduação em Imunologia Básica e Aplicada, Instituto de Ciências Biológicas, Universidade Federal do Amazonas (UFAM), Manaus, Brazil; ^2^ Diretoria de Ensino e Pesquisa, Fundação Hospitalar de Hematologia e Hemoterapia do Amazonas (HEMOAM), Manaus, Brazil; ^3^ Programa de Pós-graduação em Ciências da Saúde, Instituto René Rachou - Fundação Oswaldo Cruz (FIOCRUZ) Minas, Belo Horizonte, Brazil; ^4^ Grupo Integrado de Pesquisas em Biomarcadores, Belo Horizonte, Brazil; ^5^ Programa de Pós-graduação em Medicina Tropical, Universidade do Estado do Amazonas (UEA), Manaus, Brazil; ^6^ Programa de Pós-graduação em Ciências Aplicadas à Hematologia, UEA, Manaus, Brazil; ^7^ Laboratório de Virologia e Imunologia, Instituto Nacional de Pesquisas da Amazônia (INPA), Manaus, Brazil

**Keywords:** childhood leukemia, leukemic microenvironment, extracellular vesicles, nano-flow cytometry, biomarkers

## Abstract

Extracellular vesicles (EVs) are heterogeneous, phospholipid membrane enclosed particles that are secreted by healthy and cancerous cells. EVs are present in diverse biological fluids and have been associated with the severity of diseases, which indicates their potential as biomarkers for diagnosis, prognosis and as therapeutic targets. This study investigated the phenotypic characteristics of EVs derived from peripheral blood (PB) and bone marrow (BM) in pediatric patients with B-cell acute lymphoblastic leukemia (B-ALL) during different treatment stages. PB and BM plasma were collected from 20 B-ALL patients at three time points during induction therapy, referred to as: diagnosis baseline (D0), day 15 of induction therapy (D15) and the end of the induction therapy (D35). In addition, PB samples were collected from 10 healthy children at a single time point. The EVs were measured using CytoFLEX S flow cytometer. Calibration beads were employed to ensure accurate size analysis. The following, fluorescent-labeled specific cellular markers were used to label the EVs: Annexin V (phosphatidylserine), CD235a (erythrocyte), CD41a (platelet), CD51 (endothelial cell), CD45 (leukocyte), CD66b (neutrophil), CD14 (monocyte), CD3 (T lymphocyte), CD19, CD34 and CD10 (B lymphoblast/leukemic blast). Our results demonstrate that B-ALL patients had a marked production of EV-CD51/61^+^, EV-CD10^+^, EV-CD19^+^ and EV-CD10^+^CD19^+^ (double-positive) with a decrease in EV-CD41a^+^ on D0. However, the kinetics and signature of production during induction therapy revealed a clear decline in EV-CD10^+^ and EV-CD19^+^, with an increase of EV-CD41a^+^ on D35. Furthermore, B-ALL patients showed a complex biological network, exhibiting distinct profiles on D0 and D35. Interestingly, fold change and ROC curve analysis demonstrated that EV-CD10^+^CD19^+^ were associated with B-ALL patients, exhibited excellent clinical performance and standing out as a potential diagnostic biomarker. In conclusion, our data indicate that EVs represent a promising field of investigation in B-ALL, offering the possibility of identifying potential biomarkers and therapeutic targets.

## Introduction

B-cell acute lymphoblastic leukemia (B-ALL) is characterized by an abnormal proliferation of B lymphoblasts/leukemic cells in the bone marrow (BM), which are released into the bloodstream and extramedullary tissues, and is the most common childhood cancer in the world (1, 2). The immunological mechanisms involved in triggering or maintaining B-ALL in patients are still being investigated. Similar to other cancers, B-ALL is characterized by a complex interplay between the immune system and leukemic cells throughout progression of the disease (3). In this context, it is important to highlight that the leukemic microenvironment comprises a diverse cellular landscape. This includes leukemic cells, hematopoietic stem cells, immune cells and bone marrow stromal cells. Together, they form a singular network of intrinsic interactions that can be explored by leukemic cells to contribute to the progression of cancer (4–6).

Historically, these interactions have been shown to be modulated by several immunological mediators, including cytokines, chemokines and growth factors (7–9). In a similar way to what occurs in these molecules, recent advances in cancer biology have revealed that heterogeneous cell membrane-derived vesicles, termed extracellular vesicles (EVs), which include exosomes and microvesicles, are released in large quantities by cancer cells, acting as key mediators of cellular communication, through bioactive charges transfer, as proteins, lipids and nucleic acids (10, 11). In addition, some studies have shown that cancer cell-derived EVs are capable of transporting oncogenic factors. These factors can then be transported and internalized by surrounding cells, leading to alterations in the gene expression of recipient cells. This process can significantly impact the progression of the disease (12–14).

Although studies in ALL are scarce compared to solid tumors, EVs have been shown to play an important role in bidirectional communication between leukemic cells and bone marrow stromal cells. Leukemic EVs targeting hematopoietic stem cells and progenitors have been shown to affect the quiescence and maintenance of the hematopoietic compartment (15). On the other hand, EVs derived from endothelial cells and mesenchymal cells sustain the activities and offer a role in protecting leukemic blasts (16, 17). In the context of tumor immunity, it was also demonstrated that EVs derived from leukemic blasts inhibit the biological function of natural killer cells and effector T cells by increasing the expression of Foxp3 and the signaling of regulatory cytokines, including TGF-β and IL-10 (18, 19). Collectively, these features highlight the potential of EVs as promising biomarkers in B-ALL, since EV levels can not only predict therapeutic responses but are also easily detectable in blood via minimally invasive methods (20).

Therefore, the aim of the present investigation was to analyze the immunophenotypic profile of cell-derived EVs in the PB and BM aspirates of newly diagnosed B-ALL patients undergoing remission induction therapy. By investigating these EVs, we hope to provide insight into the use of EVs as potential biomarkers in childhood leukemia.

## Materials and methods

### Ethics statement

This study was submitted to and approved by the Ethics Committee at Fundação Hospitalar de Hematologia e Hemoterapia do Amazonas (HEMOAM), under protocol registration number #739.563. Prior to the inclusion of all the patients and controls in the study, all the respective parents or legal guardians read and signed the informed assent form. The study was carried out in accordance with the principles of the Helsinki Declaration and Resolution 466/2012 of the Brazilian National Health Council, which relates to research involving human participants.

### Patients and control subjects

The study population consisted of 20 patients under the age of 15 who had been recently diagnosed with B-ALL at Fundação HEMOAM, the reference center for diagnosis and treatment of hematological diseases in the state of Amazonas, Brazil. The diagnosis was performed according to the classification criteria and guidelines of the World Health Organization (21). The B-ALL patients were subdivided into two subgroups (B-ALL peripheral blood [PB] and B-ALL bone marrow [BM]), according to the biological material used to measure the EVs. The B-ALL PB group consisted of 10 patients (7 males and 3 females), with a median age of 3 years; IQR = 2-9. The BM group consisted of 10 patients (8 males and 2 females), with a median age of 5 years; IQR = 3-6. Additionally, 10 children without leukemia (5 males and 5 females) with a median age of 9 years, IQR = 6-13, were included as a control group. For this, only PB samples were collected to provide a reference value in the analyses, since BM aspiration is a very invasive procedure. The children recruited in this study had not experienced any infections for at least four weeks prior to the collection of samples and did not present immunological alterations in the leukocyte series. The demographic and clinical data, together with the hematological patterns of the studied population are summarized in **Tables 1**, **2**, respectively.

**Table 1:** Demographic and clinical characteristics of the study population.

Variables	CG PB (n=10)	B-ALL PB (n=10)	B-ALL BM (n=10)
**Age, median (IQR)**	9 (6-13)	3 (2-9)	5 (3-6)
**Sex, Male/Female**	5M/5F	7M/3F	8M/2F
Age group, n (%)			
1 to <5	1 (10%)	7 (70%)	4 (40%)
5 to <10	4 (40%)	1 (10%)	5 (50%)
10 to <15	5 (50%)	2 (20%)	1 (10%)
Immunophenotyping			
Common B-ALL (CD10^+^)	–	10 (100%)	10 (100%)
CNS infiltration			
Absent	–	10 (100%)	10 (100%)
Present	–	0 (0%)	0 (0%)
Cytogenetics			
Good prognosis	–	10 (100%)	10 (100%)
Poor prognosis	–	0 (0%)	0 (0%)
Risk stratification at D0			
Low Risk	–	7 (24%)	6 (27%)
High Risk	–	3 (24%)	4 (55%)
Risk re-stratification at D15			
True low risk	–	0 (0%)	1 (10%)
Low intermediate risk	–	6 (60%)	5 (50%)
High risk rapid responder	–	3 (30%)	4 (40%)
High risk slow responder	–	1 (10%)	0 (0%)
MRD at D15			
Negative	–	1 (10%)	1 (10%)
Positive	–	9 (90%)	9 (90%)
Myelogram at D35 [n (%)]			
M1	–	100 (100%)	100 (100%)
M2	–	0 (0%)	0 (0%)
M3	–	0 (0%)	0 (0%)

CG, control group; B-ALL, B-cell acute lymphoblastic leukemia; PB, peripheral blood; BM, bone marrow; IQR, interquartile range; CNS, central nervous system; MRD, measurable residual disease; D0, diagnosis baseline; D15, day 15 of induction therapy; D35, end of the induction therapy; M1, <5% lymphoblasts; M2, 5-25% lymphoblasts; M3, >25% lymphoblasts.

**Table 2:** Hematological characteristics of the study population.

Characteristics	CG PB (n = 10)	B-ALL PB (n = 10)	B-ALL BM (n = 10)	*p-value*
Total leukocytes (x10^3^/uL), median (IQR)	7,540 (6,788-8,178)	9,135 (4,593-12,195)	56,735 (41,865-76,825)	**0.0014^b^**
Lymphoblasts ABS [%], median (IQR)	–	7,239 [62%] (995- 8,278)	47,964 [82%] (28,483-64,920)	**0.0048^b^**
Neutrophils (x10^3^/uL), median (IQR)	3.24 (2.89-3.55)	0.43 (0.18-0.98)	0.30 (0.21-0.64)	**<0.0001^a^**
Lymphocytes (x10^3^/uL), median (IQR)	3.18 (2.50-3.73)	3.10 (2.25- 4.29)	3.19 (3.13-4.61)	0.7031
Monocytes (x10^3^/uL), median (IQR)	0.40 (0.29-0.54)	0.11 (0.00-0.21)	0.13 (0.05-0.27)	**0.0005^a^**
Hemoglobin (g/dL), median (IQR)	13.4 (12.3-13.7)	8.3 (3.7-9.7)	7.1 (4.6-8.1)	**0.0010^a^**
Platelets (x10^3^/uL), median (IQR)	325 (302-434)	54 (28-101)	53 (24-89)	**<0.0001^a^**

CG, control group; BM, bone marrow; PB, peripheral blood; IQR, interquartile range. Reference values: Leukocytes: 5.2 - 12.4 x10³/µL; Neutrophils: 1.9 - 8 x10³/µL; Lymphocytes: 0.9 - 5.2 x10³/µL; Monocytes: 0.16 - 1 x10³/µL; Hemoglobin: 12 - 18 g/dL; Platelets: 130 - 140 x10³/µL. Significant differences of p<0.05 are represented in bold with the following superscript letters: “a” and “b”, which refer to comparisons of the B-ALL PB group with the CG and B-ALL BM group, respectively.

### Treatment regimen

All the B-ALL patients underwent remission induction therapy (according to the protocol and guidelines found in the Brazilian Group for Treatment of Childhood Leukemia, version 2009), which is an intensive stage of chemotherapy of fundamental importance for the prognosis of patients, and whose objective is to achieve disease remission, with less than 5% lymphoblasts in five weeks. The treatment regimen includes the drugs prednisone, dexamethasone, vincristine, daunorubicin, L-asparaginase and MADIT (intrathecal methotrexate, cytarabine and dexamethasone) (22).

### Biological sample collection

The PB and BM samples of the B-ALL patients were obtained by venipuncture and iliac crest aspiration, respectively, at three consecutive time points, referred to as: D0 (diagnosis baseline), D15 (day 15 of induction therapy) and D35 (end of the induction therapy). In addition, PB samples from controls were obtained (single time point) via venipuncture. After collection, the biological samples were transferred to EDTA vacuum tubes (BD Vacutainer^®^ EDTA K2) and submitted to centrifugation at 600 *x*g, for 10 minutes at room temperature (RT). Subsequently, the supernatants or platelet-poor plasma were collected and immediately stored at -80°C until processing for EV measurement.

### Sample preparation and extracellular vesicle measurement via flow cytometry

**Figure 1:**
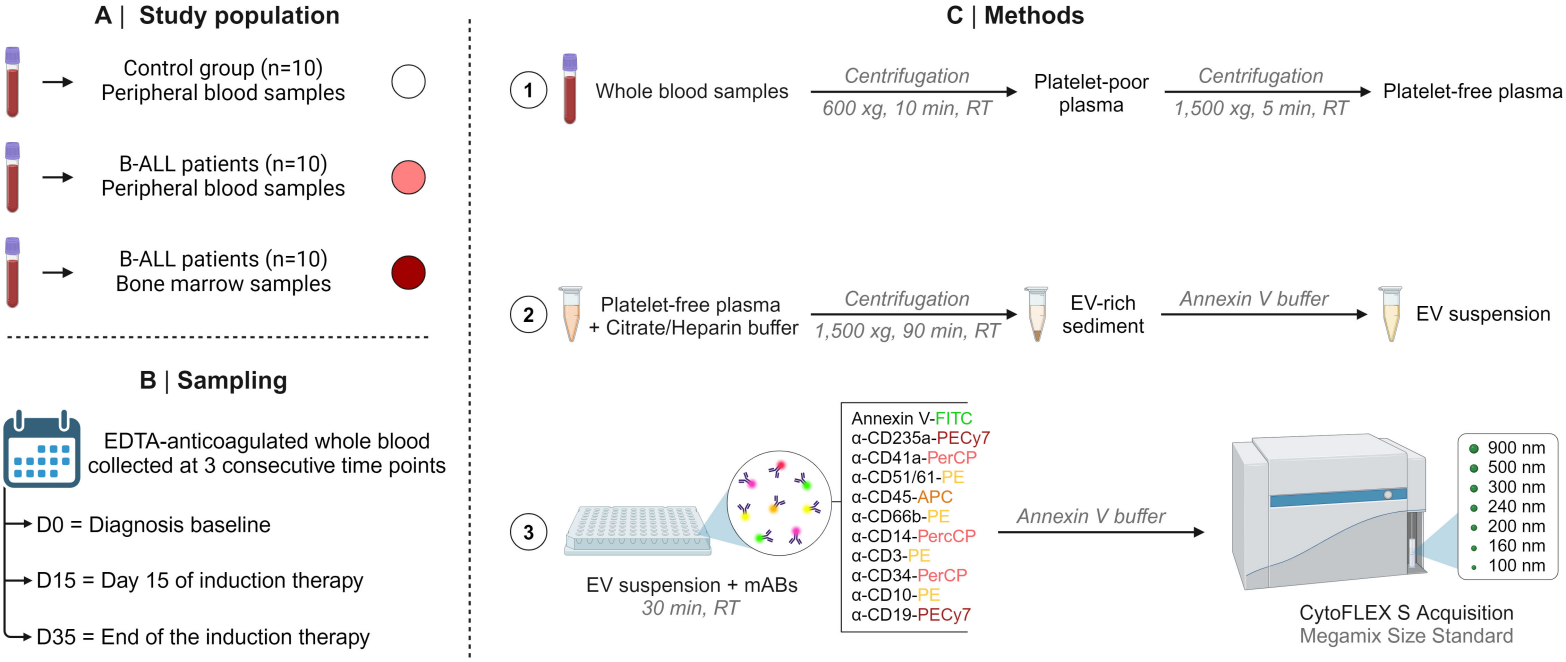
Compendium of study. The study population **(A)**, sampling **(B)**, and methods **(C)**, including the steps of the protocol are summarized in this figure.

Initially, the samples were thawed at 37 °C and then centrifuged at 1,500 *x*g for 5 minutes to obtain platelet-free plasma. The latter was diluted in a citrate buffer solution containing heparin (1 μg/mL) and centrifuged at 1,500 *x*g for 90 minutes at RT. The EV-rich sediment was resuspended in commercially available Annexin V buffer (25 mM CaCl_2_ solution in 140 mM NaCl and 10 mM HEPES, pH 7.4; BD Bioscience, San Diego, CA, USA) to obtain the EV suspension. Aliquots of 100 μL of EV suspension were transferred to a plate containing 2 μL of distinct monoclonal antibodies (mAB) to evaluate the immunophenotypes of the study panel. Of importance, prior to staining, mABs were centrifuged at 1,500 x g for 30 minutes to remove fluorescent particles. The panel was composed of specific markers of B cell lineage and maturation stage, which are used for the diagnosis and monitoring of B-ALL. Markers of cellular populations/elements (erythrocytes, platelets and leukocytes) were also used, which are frequently used as parameters for classifying therapeutic response. Thus, the study panel was composed of CD235a (erythrocyte), CD41a (platelets), CD51 (endothelial cell), CD45 (leukocytes), CD66b (neutrophils), CD14 (monocytes), CD3 (T lymphocytes), CD19 (B lymphocyte/B lymphoblast) and CD34 and CD10 (B lymphoblast/leukemic blast); and 2.5 μL of Annexin V-FITC, which binds to phosphatidylserine residues expressed on the surface of EVs. Internal autofluorescence control was included in each trial run, in which an aliquot of EV suspension was incubated in the absence of mAB and Annexin V-FITC (all purchased from BD Bioscience, San Diego, CA, USA). Additionally, aliquots of mAB and Annexin V-FITC, incubated in the absence of EVs, were also used as internal controls. After incubation for 30 minutes in the dark at RT, 300 μL of Annexin V buffer was added to the wells of each plate and then transferred to FACS tubes. The samples were acquired in a flow cytometer (CytoFLEX S, Beckman Coulter, Brea, CA, USA) with volume control aspirated per minute. The CytoFLEX S has a volumetric sample injection system that allows counting of absolute particles. The sample flow rate was 30 μL/min, and the sample acquisition occurred during 2 minutes per sample. Calibration beads (Megamix-Plus FSC and SSC, Biocytex, Marseille, France) with standard sizes of 100, 160, 200, 240, 300, 500, 900 nm were used to identify different EV size ranges, defined as: small EVs (sEVs): 100-200 nm; medium EVs (mEVs): 201-500 nm; and large EVs (lEVs): 501-900 nm. The steps of the protocol are summarized in **Figure 1**. Different gating strategies were used to analyze the phenotypic characteristics and size of the EVs, according Megamix beads, as illustrated in **Supplementary Figures 1**, **2**.

### Conventional statistical analysis

The comparative analysis between the B-ALL patients and controls was carried out using Student’s t test or the Mann-Whitney test. Comparisons among the timepoints of induction therapy (D0, D15 and D35) and EV size ranges (sEVs, mEVs and lEVs) were performed using one-way ANOVA followed by the Tukey or Friedman tests followed by Dunn’s test; along with the paired t test or Wilcoxon matched-pairs signed-ranks test. In all cases, the Shapiro-Wilk test was used to verify the normality of the data and significance was considered when p was <0.05. The GraphPad Prism software v8.0.2 (San Diego, CA, USA) was used for statistical analysis.

### Overall signatures of extracellular vesicles

The signature analysis was carried out according to Kerr et al. (2021) (23), by converting the original results of each variable expressed as a continuous variable in categorical data. For this purpose, the global median values obtained for the whole data universe from all participants (B-ALL patients on different days of induction therapy and the controls) as the cut-off to classify the patients with low (below the cut-off) or high (above the cut-off) production of EVs. The following cut-offs were used: (EV-CD235a^+^ = 27,917; EV-CD41a^+^ = 658; EV-CD51/61^+^ = 4,839; EV-CD45^+^ = 296; CD66b^+^ = 797; EV-CD14^+^ = 200; EV-CD3^+^ = 445; EV-CD34^+^ = 299; EV-CD10^+^ = 3,289; and EV-CD19^+^ = 7,617) expressed as an absolute number of EVs/mm^3^ of plasma. The overall signatures were assembled in radar charts using the 50^th^ percentile as a threshold to identify the proportion of subjects with EV populations above the global median cut-off.

### Biological networks of extracellular vesicles

Analysis of correlation networks was performed to evaluate the multiple associations among the EV populations in the B-ALL patients and the controls. The association between the EV levels was determined by using the Spearman correlation coefficient in GraphPad Prism, v8.0.2 (GraphPad Software, San Diego, CA, USA), and statistical significance was considered only if p was <0.05. After performing the correlation analysis between EV populations, a database was created using Microsoft Excel^®^ program. Then, the significant correlations were compiled using the open source Cytoscape software, v3.9.1 (National Institute of General Medical Sciences, Bethesda, MD, USA). The biological networks were constructed using circular layouts in which each EV population is represented by a globular node, in which the larger the nodule size, the greater the number of correlations established. The correlation indices (r) were used to categorize the correlation strength as negative (r <0), moderate (0.36≥ r ≤0.68), or strong (r >0.68), which were represented by connecting edges, as proposed by Taylor (1990) (24). Cytoscape software and Microsoft PowerPoint program were used for the graphics.

### Fold change and performance analysis of extracellular vesicles

The magnitude of change in the EV levels in the B-ALL patients was calculated as the proportion ratio between the serum levels observed for each B-ALL patient at the diagnosis baseline (D0) divided by the median values reported for the control group. The magnitude of changes in the EV levels in the PB were determined considering decreased (≤ 1x) and increased (≥ 1x) levels in relation to the median values observed in the control group. Bubble charts were generated using Microsoft Excel^®^. Receiver operating characteristic (ROC) curve analysis (25) was carried out to assess the performance of EVs as biomarkers for B-ALL in the study population. ROC curve data were used to define cut-off points for the EVs evaluated. Performance indices of sensitivity (Se), specificity (Sp) and likelihood ratio (LR) were calculated at a specific cut-off and the area under the curve (AUC) and p-value were considered as indicators of global accuracy. The MedCalc v7.3.0 (Ostend, West Flemish, BE) and GraphPad Prism software v8.0.2 (San Diego, CA, USA) were used for statistical analysis and construction of the ROC curves.

## Results

### Characterization of the profiles of the extracellular vesicles at diagnosis baseline

**Figure 2:**
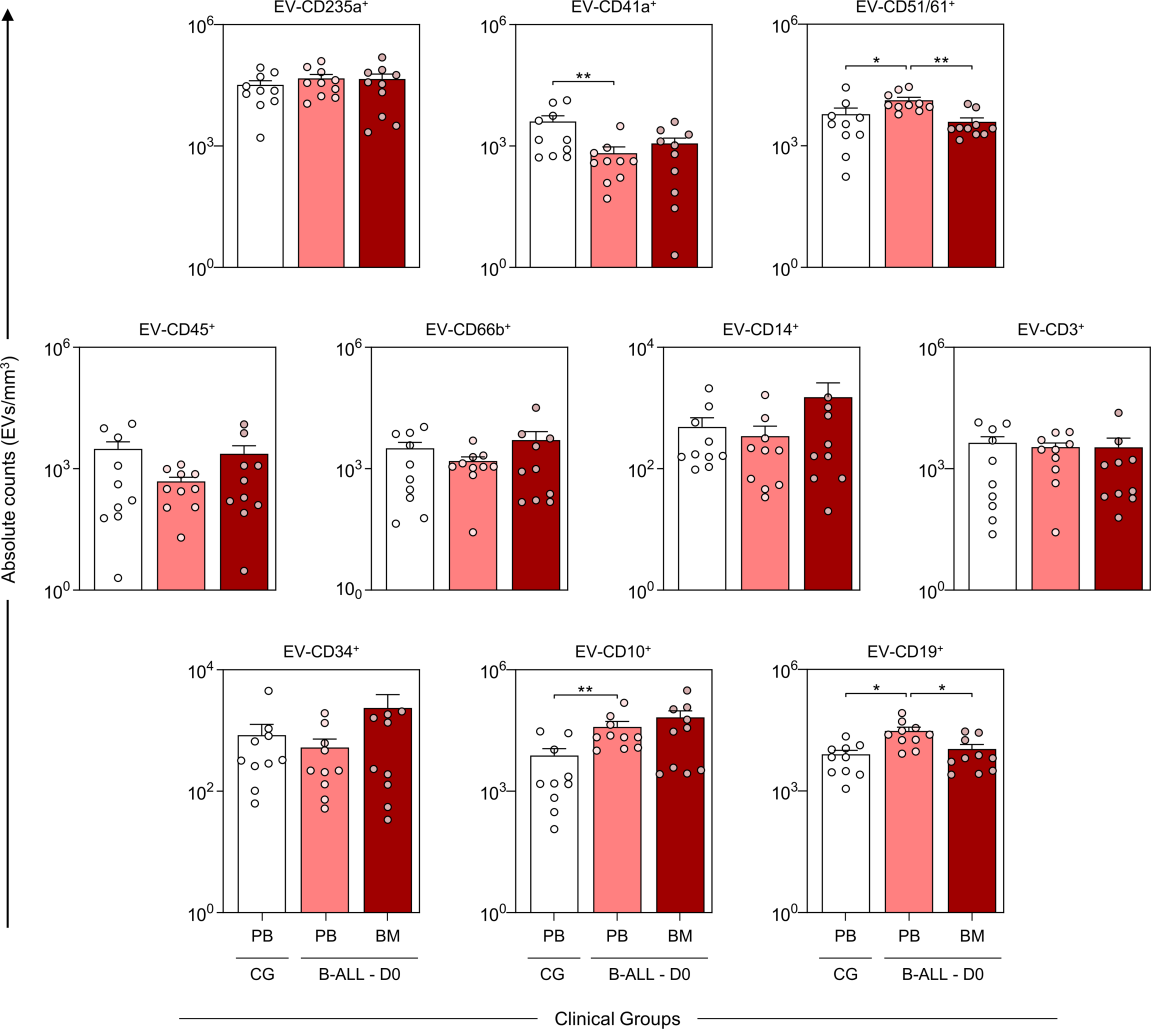
Characterization of the profile of the extracellular vesicles at diagnosis baseline. The EV populations were measured at the time of diagnosis in the B-ALL PB (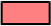) and B-ALL BM (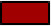) groups and in the control group (CG) (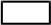). The count and immunophenotypic characterization of EVs was performed using flow cytometry, as described in the Materials and Methods section. The results are presented using bar and symbol charts, reported in log10 scale, showing the mean with standard error of the absolute number of EVs/mm^3^ of plasma. Statistical analyses were performed using Student’s t test or the Mann-Whitney test and significant differences are highlighted by asterisks for p<0.01 (**) or p<0.05 (*).

The characterization of the EV profile at diagnosis (D0) demonstrated that the B-ALL PB group had a decrease in platelet-derived EVs (EV-CD41a^+^) and an increase in endothelial cell-derived EVs (EV-CD51/61^+^) and B lymphoblasts/lymphocytes with CD10 and CD19 phenotype (EV-CD10^+^ and EV-CD19^+^) when compared to control group. Additionally, an increase in the levels of EV-CD51/61^+^ and EV-CD19^+^ was observed when compared to the B-ALL BM group. Nevertheless, a thorough analysis revealed a trend towards increased levels of leukocyte-derived EVs (EV-CD45^+^), neutrophils (EV-CD66b^+^), monocytes (EV-CD14^+^) and B lymphoblast with the CD34 phenotype (EV-CD34^+^) in the B-ALL BM group (**Figure 2**).

### Kinetics of extracellular vesicles during induction therapy

**Figure 3:**
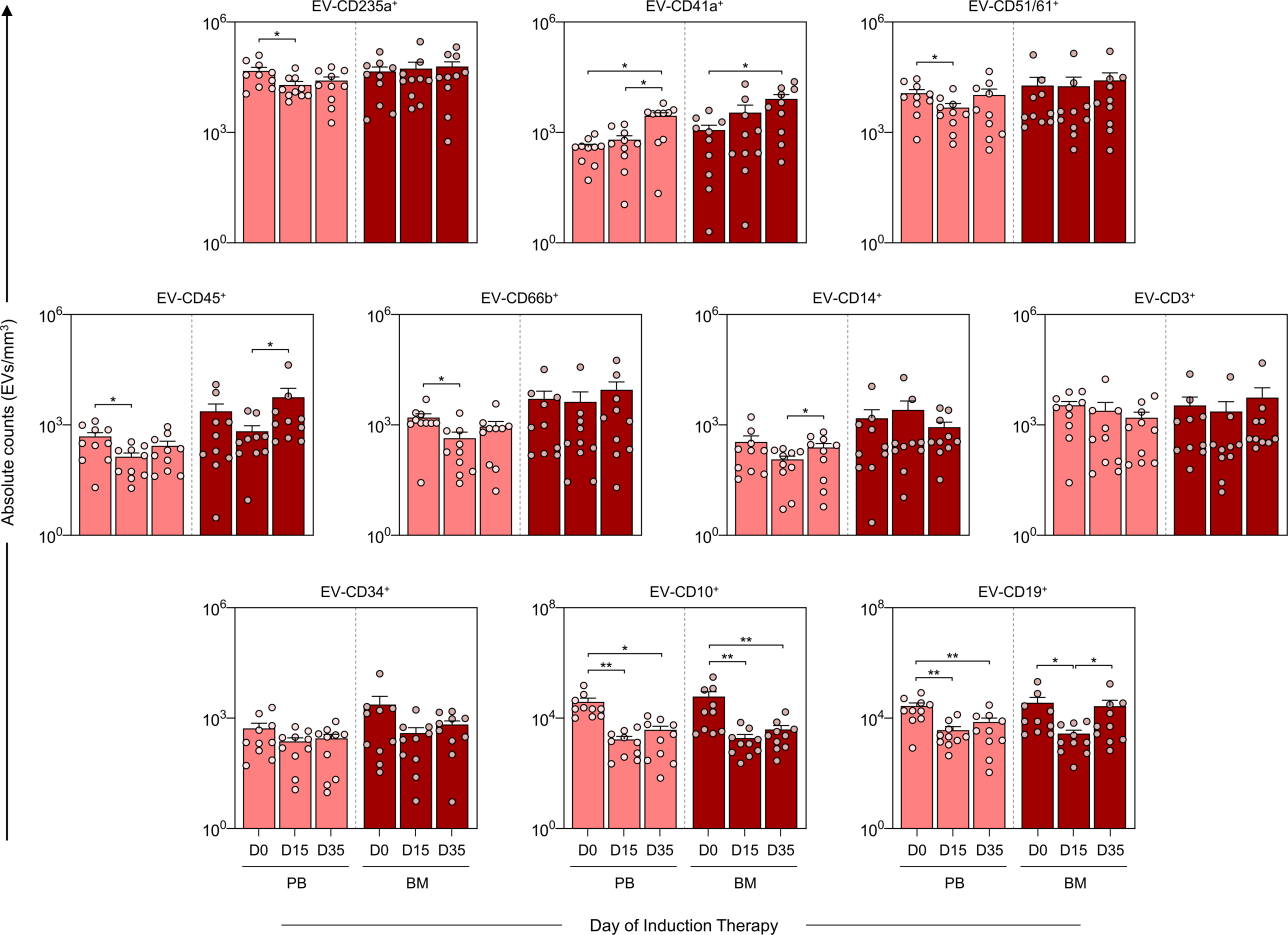
Kinetics of extracellular vesicles during induction therapy. The EV populations were measured on D0, D15, and D35 in the B-ALL PB (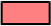) and B-ALL BM (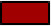) groups to assess the behavior of these EVs during remission induction therapy. The count and immunophenotypic characterization of the EVs was performed using flow cytometry, as described in the Materials and Methods section. The results are presented using bar and symbol charts, reported in log10 scale, showing the mean with standard error of the absolute number of EVs/mm^3^ of plasma. Statistical analyses were performed using a paired t test or Wilcoxon matched-pairs signed-rank test for comparisons between D0, D15 and D35 and significant differences are highlighted by asterisks for p<0.01 (**) or p<0.05 (*).

The analysis of EV kinetics of in PB and BM was performed on the samples from D0, D15 and D35 to assess the EV levels on diagnosis, at the beginning and at the end of induction therapy (**Figure 3**). The results demonstrated a decrease in EV-CD235a^+^, EV-CD51/61^+^, EV-CD45^+^ and EV-CD66b^+^ in the PB group on D15. Moreover, there was a noticeable decline in EV-CD10^+^ and EV-CD19^+^ on D15 and D35. This trend was similarly observed in the B-ALL BM group, wherein EV-CD10^+^ decreased at D15 and D35. However, EV-CD19^+^ exhibited a distinct pattern, decreasing on D15 and increasing on D35. Furthermore, both the B-ALL PB and B-ALL BM groups showed an increase in EV-CD41a^+^ on D35. In addition, a specific an increase in EV-CD14^+^ and EV-CD45^+^ was observed in the B-ALL PB and B-ALL BM groups, respectively.

### Kinetics of extracellular vesicles according to size range

**Figure 4:**
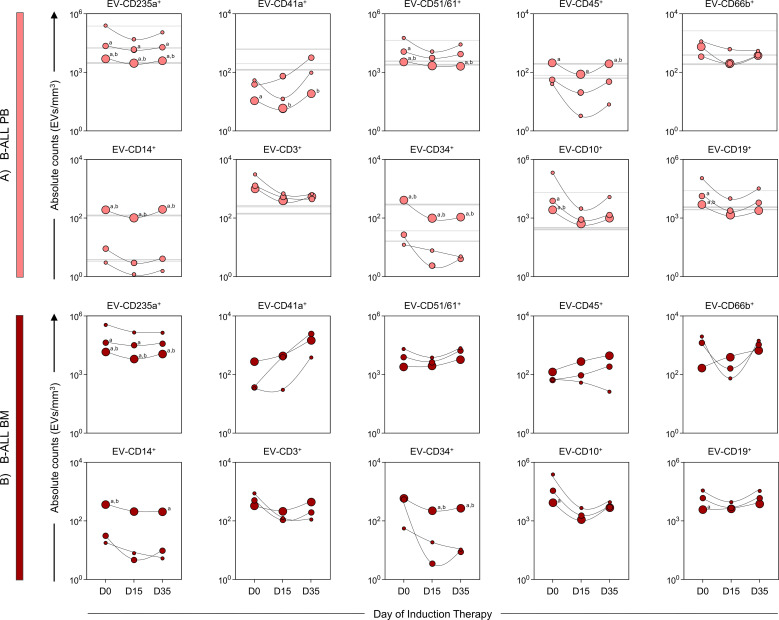
Kinetics of extracellular vesicles according to size range. The EV populations were analyzed in **(A)** B-ALL PB (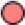) and **(B)** B-ALL BM (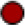) groups according to their size, based on Megamix beads size range, being divided into small EVs = 100-200 nm (sEVs), medium EVs = 201-500 nm (mEVs) and large EVs = 501-900 nm (lEVs), represented by the symbols: “
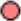
”, “
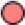
” and “
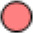
”, respectively. For the control group, sEVs, mEVs, and lEVs were represented by gray background lines: “
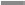
”, “
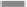
” and “
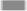
”, respectively. The count, size and immunophenotypic characterization of the EVs was performed using flow cytometry, as described in the Materials and Methods section. The results are presented using symbol charts, reported in log10 scale, showing the median of the absolute number of EVs/mm^3^ of plasma. Statistical analyses were performed using a paired t test or Wilcoxon matched-pairs signed-rank test for comparisons between sEVs, mEVs, and lEVs and significant differences are represented by the letters: “a” and “b”, which refer to the comparisons with sEVs and mEVs, respectively.

To better understand the size distribution of the EV populations evaluated, we used calibration beads with specific sizes (100, 160, 200, 240, 300, 500, 900 nm). Based on this calibration, we classified the EVs into three size ranges: small EVs (sEV: 100-200 nm), medium EVs (mEV: 201-500 nm) and large EVs (lEV: 501-900 nm) (**Figure 4**). On diagnosis baseline and throughout induction therapy (D0, D15 and D35) in the B-ALL PB group, there was a consistent predominance of EV-CD235a^+^ and EV-CD51/61^+^ in the sEV and mEV size ranges. In contrast, the EV-CD45^+^, EV-CD14^+^, EV-CD34^+^ populations showed a predominance of lEV during the treatment. The EV-CD41a^+^ population showed an increase in sEV on D0 compared to control group, followed by an increase in mEV on both D15 and D35, compared to lEV. In addition, EV-CD10^+^ and EV-CD19^+^ exhibited a predominance of sEV, followed by mEV on D0 (**Figure 4A**). In the B-ALL BM group, EV-CD235a^+^ predominated in both sEV and mEV ranges, while EV-CD14^+^ and EV-CD34^+^ exhibited a predominance of lEV. On D0, the EV-CD10^+^ and CD19^+^ populations showed an increase in sEV compared to lEV (**Figure 4B**).

### Signature of extracellular vesicles during induction therapy

**Figure 5:**
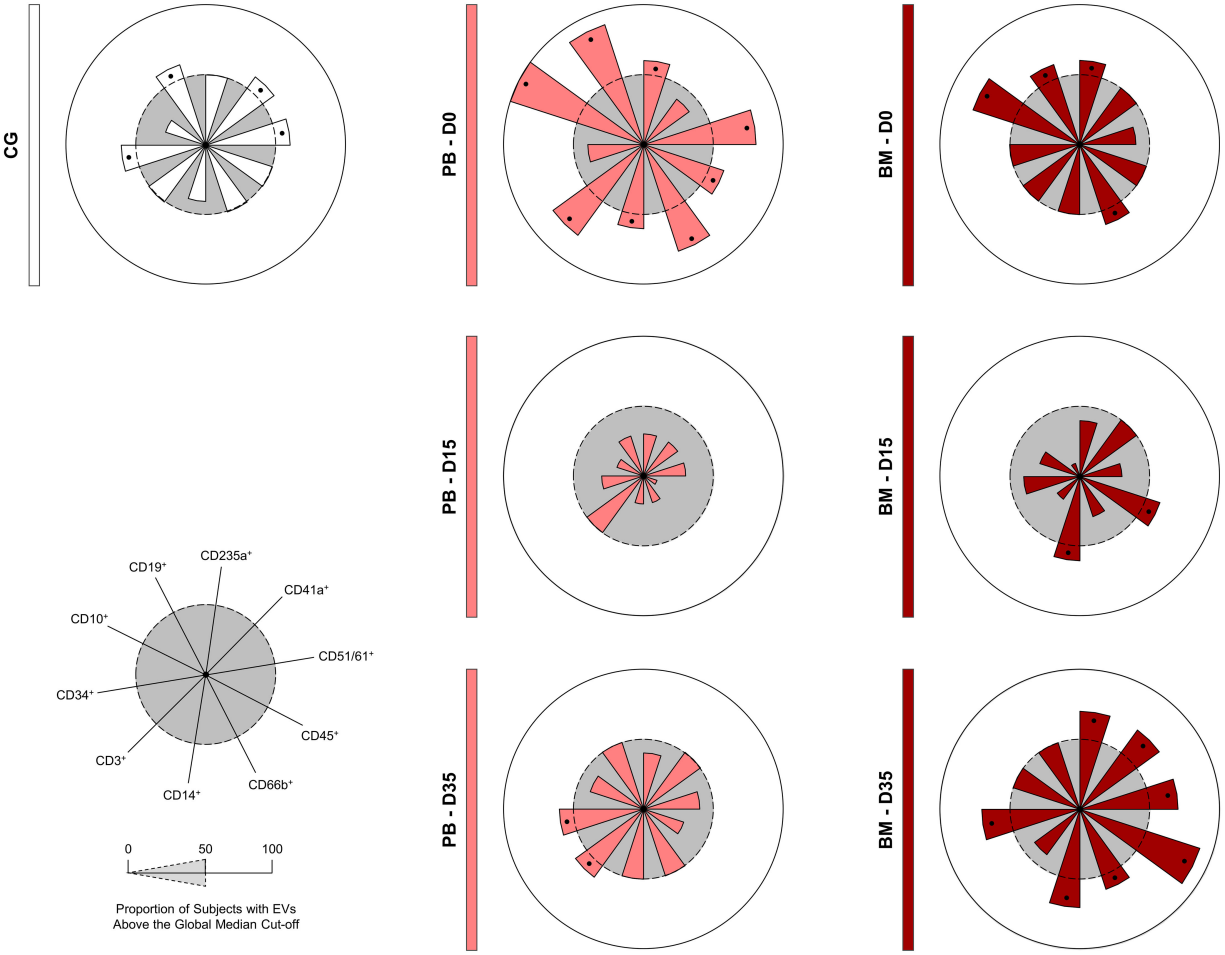
Signature of extracellular vesicles during induction therapy. The overall signature of EV populations in the B-ALL patients was assembled on D0, D15 and D35. Data, originally expressed as absolute number of EVs/mm^3^ of plasma, were converted into categorical data using the global median values, which were used as a cut-off point to classify the study population as being a low or high producer of the EVs evaluated. The overall signatures were assembled in radar charts using the 50^th^ percentile as the threshold (central circle/gray zone) to identify EV populations with increased levels in a higher proportion of patients. Cellular markers: CD235a (erythrocyte), CD41a (platelet), CD51 (endothelial cell), CD45 (leukocyte), CD66b (neutrophil), CD14 (monocyte), CD3 (T lymphocyte), CD34 and CD10 (B lymphoblast/Leukemic blast) and CD19 (B lymphocyte/B lymphoblast).

To further refine the characterization of the EV profile in the B-ALL patients (**Figure 5**), we calculated the median for each EV population across all the patients. This median value was then used as a cut-off to categorize patients as low or high producers of specific EVs. Our findings demonstrated that on D0, compared to the control group, the B-ALL PB group displayed a greater production of most EV populations, except for EV-CD41a^+^ and EV-CD34^+^. On D15, there was a significant decrease in the production of all EV populations. By D35, only EV-CD3^+^ was observed and EV-CD14^+^ remained elevated. In contrast, the B-ALL BM group exhibited a different pattern. On D0, high production was observed for EV-CD235a^+^, EV-CD66b^+^, EV-CD10^+^ and EV-CD19^+^. By D15, only EV-CD45^+^ and EV-CD14^+^ showed an increase. Nonetheless, on D35, there was an increase in the production of most EV populations, except EV-CD3^+^, EV-CD10^+^ and EV-CD19^+^.

### Biological network of extracellular vesicles during induction therapy

**Figure 6:**
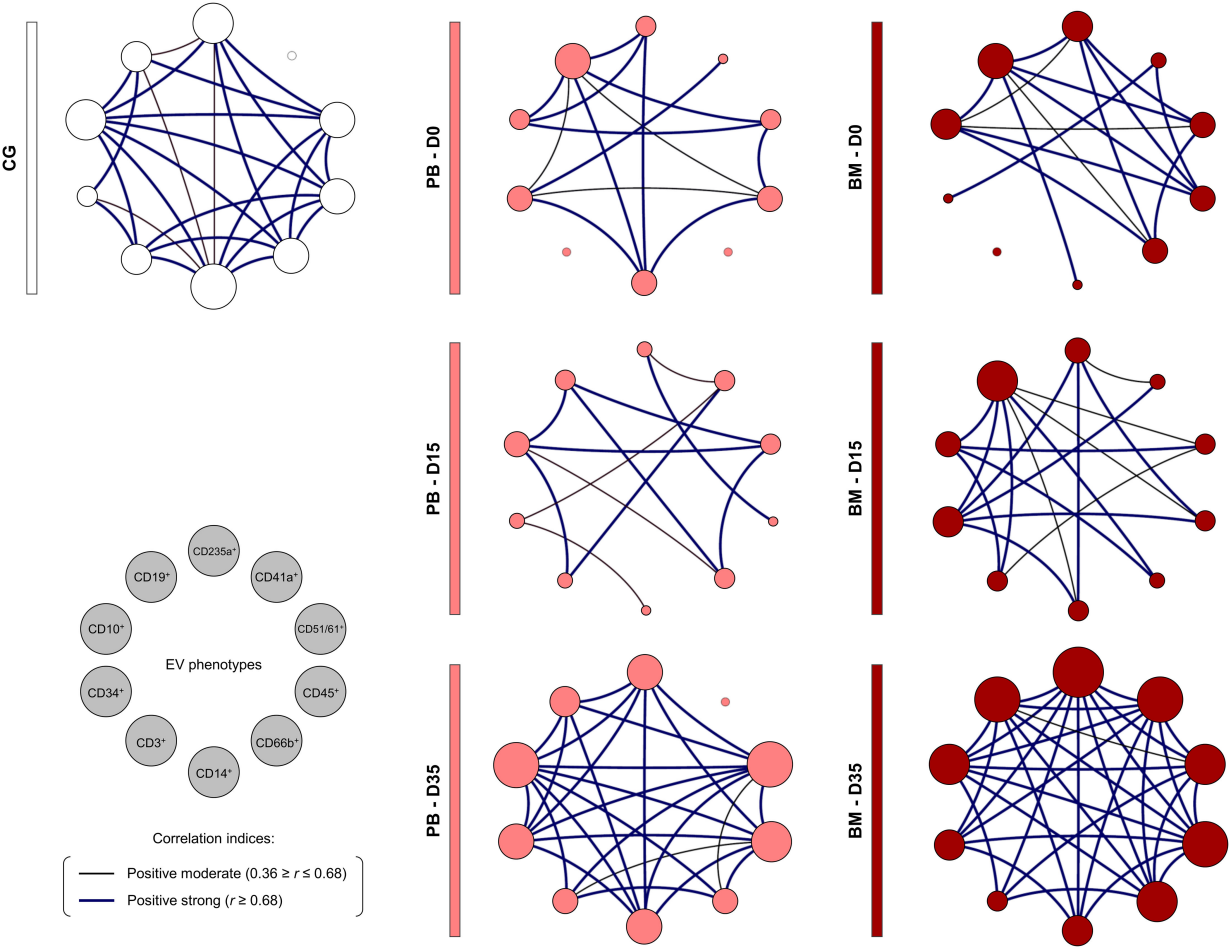
Biological network of extracellular vesicles during induction therapy. Integrative networks were assembled to identify the complex interactions among EV populations during induction therapy. Colored nodes are used to identify the EVs in the B-ALL PB (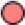) and B-ALL BM (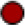) groups and in the control group (CG) (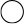), where the larger the node, the greater the number of interactions established. Correlation analysis was employed to construct integrative networks according to significant “r” scores at p<0.05 using the Spearman correlation test. Connecting edges illustrate the positive correlations between pairs of attributes, according to the strength of correlation as described in the Materials and Methods section. Different colored and thickness are used to represent moderate correlations (black fine edges) and strong correlations (dark blue solid edges). Cellular markers: CD235a (erythrocyte), CD41a (platelet), CD51 (endothelial cell), CD45 (leukocyte), CD66b (neutrophil), CD14 (monocyte), CD3 (T lymphocyte), CD34 and CD10 (B lymphoblast/Leukemic blast) and CD19 (B lymphocyte/B lymphoblast).

The construction of integrative biological networks was performed to assess the complex interactions between EV populations during induction therapy (**Figure 6**). The results demonstrated that the B-ALL PB group exhibited a network with a restricted number of interactions on D0. On D15, a minor decrease in the number of neighborhood connections was observed. Despite this, on D35, there was a substantial increase in the number of interactions between EV populations, resulting in a network with a profile that was more similar to the control group. Similarly, the B-ALL BM group’s network displayed a restricted number of interactions on D0 when compared to the control group. This number increased slightly on D15, followed by a significant increase in interactions between EV populations on D35.

### Fold change and performance of extracellular vesicles CD10^+^and CD19^+^ as diagnostic biomarkers of B-ALL

**Figure 7:**
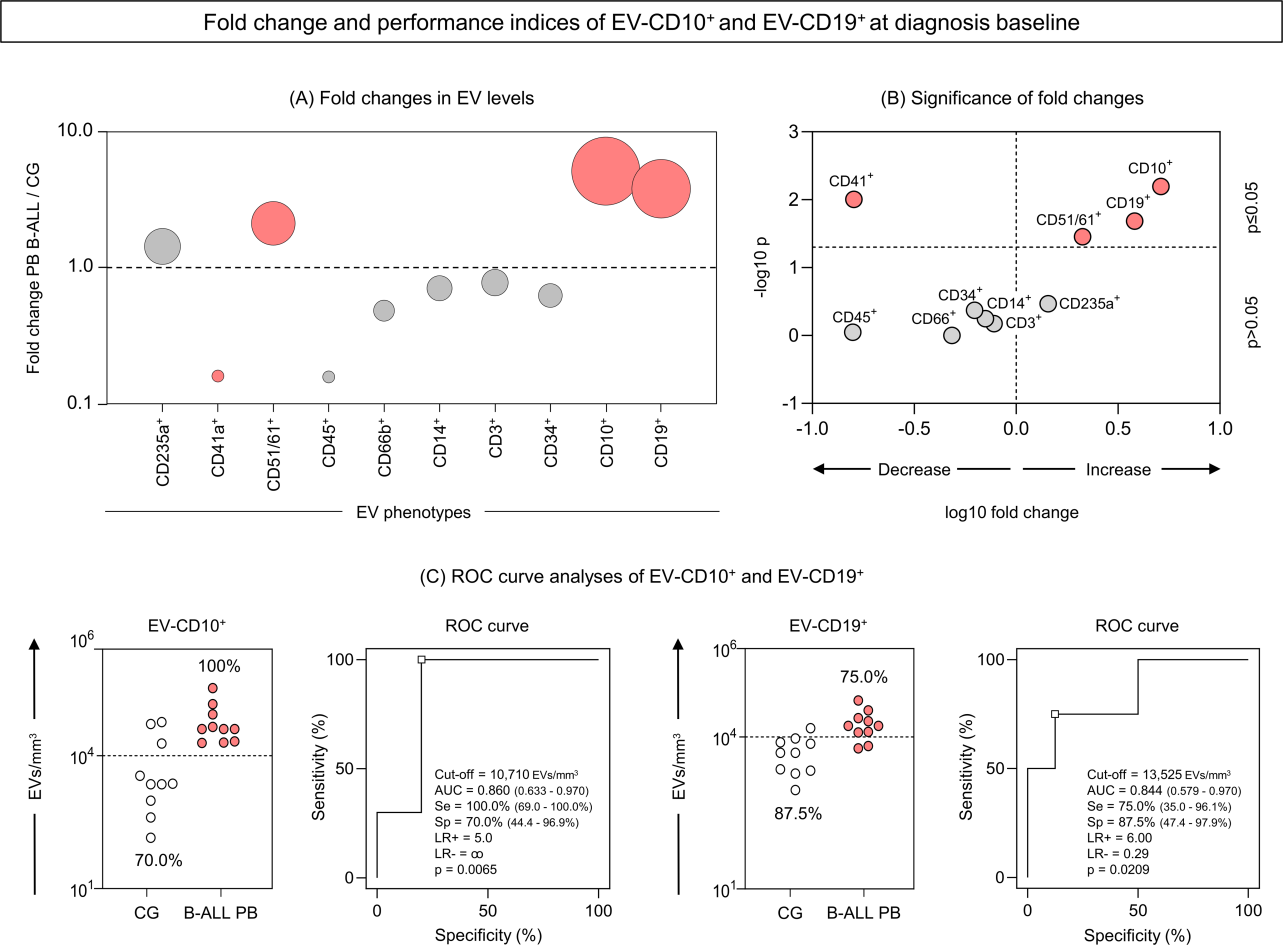
Fold change and performance of the extracellular vesicles CD10^+^ and CD19^+^ as diagnostic biomarkers of B-ALL. The fold changes **(A)** and significance of fold changes **(B)** were performed in the peripheral blood of the B-ALL patients at the diagnosis baseline as described in the Materials and Methods section. Receiver operating characteristic (ROC) curve analysis was carried out to assess the performance of EV-CD10^+^ and EV-CD19^+^ levels as diagnostic biomarkers for B-ALL **(C)**. ROC curves were assembled to define the cut-off points and calculate the following performance indices: sensitivity (Se), specificity (Sp), likelihood ratio (LR), the best cut-off point, as well as the area under the curve (AUC) and p-value as indicators of global accuracy, as described in the Materials and Methods section.

To identify potential diagnostic biomarkers, we performed a translational analysis that focused on EV-CD10^+^ and EV-CD19^+^ levels measured in the PB of the B-ALL patients at D0. Our findings revealed significant changes in EV levels in the B-ALL patients when compared to the control group at D0. EV-CD10^+^ levels showed the most dramatic increase (over 5-fold), followed by EV-CD19^+^ (over 3.5-fold) and EV-CD51/61 (over 2-fold). In addition, EV-CD41a exhibited a significant decrease (below 1.5-fold) (**Figures 7A, B**). To assess the diagnostic potential of these EV levels in the B-ALL patients, we performed ROC curve analysis. This analysis calculates the area under the curve (AUC), a measure of overall accuracy, along with sensitivity (Se), specificity (Sp) and likelihood ratio (LR) to evaluate how well an EV level discriminates B-ALL from the control group. Data analysis demonstrated that EV-CD10^+^ showed high performance (Se = 100.0% and Sp = 70.0%) and good global accuracy (AUC = 0.860 and p = 0.0065) to discriminate the B-ALL PB group from the control group. However, EV-CD19^+^ levels exhibit a moderate performance (Se=75.0% and Sp=87.5%) and global accuracy (AUC = 0.844 and p = 0.0209) (**Figure 7C**). Additionally, EV-CD41a and EV-CD51/61 also presented moderate/high performance (Se = 66.7% and Sp = 100.0%/Se = 100.0% and Sp = 75.0%, respectively) and global accuracy (AUC = 0.852 and p = 0.0118/AUC = 0.812 and p = 0.0357, respectively) (**Supplementary Figure 3**).

### Profile, Kinetic, fold change and performance of extracellular vesicles CD10^+^CD19^+^ as diagnostic biomarkers of B-ALL

**Figure 8:**
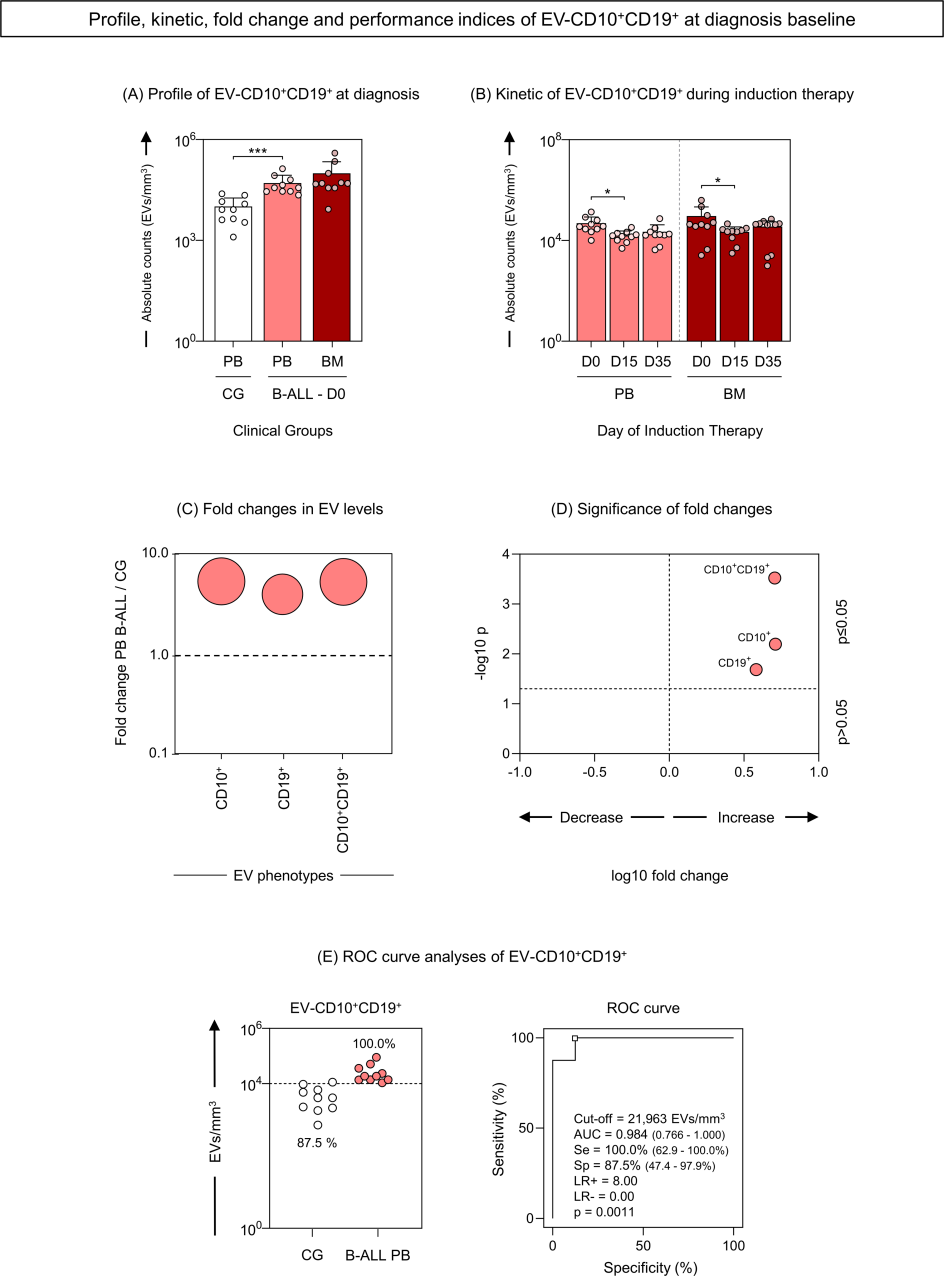
Profile, kinetic, fold change and performance of extracellular vesicles CD10^+^CD19^+^ as diagnostic biomarkers of B-ALL. The EV-CD10^+^CD19^+^ (double-positive) populations were analyzed at the diagnosis baseline **(A)** and during induction therapy **(B)** in the B-ALL PB (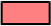) and B-ALL BM (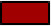) groups and in the control group (CG) (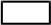). The count and immunophenotypic characterization of EV-CD10^+^CD19^+^ was performed using flow cytometry. The results are presented using bar and symbol charts, reported in log10 scale, showing the mean with standard error of the absolute number of EVs/mm^3^ of plasma. Statistical analyses were performed using the Mann-Whitney test or Wilcoxon matched-pairs signed-rank test and significant differences are highlighted by asterisks for p<0.001 (***) or p<0.05 (*). The fold changes **(C)** and significance of fold changes **(D)** were performed in the peripheral blood of the B-ALL patients at the diagnosis baseline. Receiver operating characteristic (ROC) curve analysis **(E)** was carried out to assess the performance of EV-CD10^+^CD19^+^ plasma levels as diagnostic biomarkers for B-ALL. ROC curves were assembled to define the cut-off points and calculate the following performance indices: sensitivity (Se), specificity (Sp), likelihood ratio (LR), the best cut-off point, as well as the area under the curve (AUC) and p-value as indicators of global accuracy.

Aiming of investigating whether CD10^+^ and CD19^+^ markers were present simultaneously in EVs, we carried out a strategy to evaluate EV-CD10^+^CD19^+^ (double-positive) in our study population. Compilation of data relating to the EVs profile; kinetics during induction therapy; fold change analysis; and performance of EV-CD10^+^CD19^+^ as biomarkers, are represented in **Figure 8**. The results demonstrated that B-ALL patients showed a significant increase in serum levels of EV-CD10^+^CD19^+^ compared to GC (**Figure 8A**). During induction therapy, a decline in D15 was observed in both the B-ALL PB and the B-ALL BM groups (**Figure 8B**). Regarding the fold change analysis, it was observed that B-ALL PB showed a pronounced increase in EV-CD10^+^CD10^+^ levels (more than 5 times) (**Figures 8C, D**). In parallel, the ROC curve analysis on D0 revealed excellent performance (Se = 100.0% and Sp = 87.5%) and global accuracy (AUC = 0.984 and p = 0.0011) to discriminate the B-ALL PB patients from the CG (**Figure 8E**).

## Discussion

Growing evidence shows that the bone marrow (BM) microenvironment plays a crucial role in the survival of leukemic blasts and that communication between these cancer cells and surrounding cells can be mediated by various soluble immune molecules such as cytokines, chemokines and growth factors (5, 8, 9). Much like what occurs in other signaling molecules, recent advances in cancer biology have revealed that EVs are released in large quantities by cancer cells. These EVs act as key mediators in cell communication, carrying bioactive loads capable of reprogramming stromal and immune cells, thereby creating a favorable microenvironment for leukemic survival and progression (11). In this study, we analyzed the profile of leukemic blast-derived EVs (EV-CD34^+^/CD10^+^/CD19^+^) in the peripheral blood (PB) and BM plasma of pediatric patients with B-ALL (B-ALL PB and B-ALL BM, respectively), at diagnosis baseline (D0) and during induction therapy (D15 and D35). Of interest, we also analyzed the levels of erythrocyte-derived EVs (EV-CD235a^+^), platelets (EV-CD41a^+^) and endothelial cells (EV-CD51/61^+^), as well as leukocyte-derived EVs (EV-CD45^+^), neutrophils (EV-CD66b^+^), monocytes (EV-CD14^+^), T lymphocytes (EV-CD3^+^) and B lymphocytes (EV-CD19^+^).

EVs contained in blood mainly originate from platelets and erythrocytes and account for about 50% of the total vesicles in healthy subjects (26). In our data, we detected a decrease in EV-CD41a^+^ levels in the B-ALL PB group compared to the control group (**Figure 2**), which reflects the intense thrombocytopenia observed in the blood count on D0 (**Table 2**). However, on D35, there was an increase in EV-CD41a^+^, indicating recovery of thrombopoiesis with increased platelet production (**Figure 3**). In the context of solid tumors, platelets are reported to play a role in the mechanisms by which cancer cells can accelerate their growth rate and evade the immune system (27–29). However, studies investigating the role of platelets in hematological malignancies are scarce (30–32). From a therapeutic point of view, it is considered that platelet count can be used as a parameter for prognostic assessment of ALL patients during and after induction therapy (33, 34). These questions highlight the need for in-depth investigations into the interactions of EV-CD41a^+^ with leukemic blasts, as well as its use as a biomarker related to thrombopoiesis or recovery of normal hematopoiesis.

The fraction of EVs derived from endothelial cells (EV-ECs) is relatively low in physiological conditions but is highly increased in pathologies characterized by endothelial dysfunction, such as thrombotic thrombocytopenic purpura, diabetes or hypertension (35). When the release of EVs derives from activated ECs, their action has been frequently associated with inflammatory processes and procoagulant states (36, 37). Our results identified high levels of EV-CD51/61^+^ on D0 in the B-ALL PB group when compared to the control group (**Figure 2**). These findings are important as they indicate greater activation of the endothelium in leukemia, which may be associated with an increased risk of thrombosis. Importantly, venous thromboembolism is described as a serious and relatively common condition in pediatric ALL patients (38, 39), and reported incidences vary from 1.1% to 36.7% (40, 41). Mechanisms underlying the increased risk are not completely understood, but studies have shown that besides treatment components, the malignancy itself can contribute to a prothrombotic state (42, 43). In this scenario, EV-ECs emerge as a potential contributor to these events since they are one of the EV populations with the most pronounced coagulation activity. This is a feature that is due to the high expression of active tissue factor, which is the main initiator of the coagulation cascade reactions (44, 45).

Not less important, pro-angiogenic effects of EV-ECs were also reported and considered to be a potential mechanism that leads to neovascularization (46, 47). One recent study demonstrated that the secretion of EC-derived EVs containing angiopoietin like 2 (ANGPTL2) played important roles in the development of murine B-ALL, sustaining leukemogenic activities of leukemic blasts (16). Collectively, these data indicate that EV-ECs actively participate in inflammation, coagulation and angiogenesis. This functional repertoire introduces the possibility of using EV-ECs as biomarkers and therapeutic targets in cancer; however, the field remains very obscure and requires further investigations, especially in the context of acute leukemias.

Regarding markers associated with leukemic blasts, our B-ALL patients showed an increase in EV-CD10^+^ and EV-CD19^+^ (**Figure 2**). CD19 is a signal amplifying coreceptor expressed throughout B-cell development, though not in the mature plasma cell stage; it is, however, the single best clinical marker for B-cell identity (48). Instead, CD10, also known as common acute lymphoblastic leukemia antigen (CALLA), is a type II cell surface integral membrane protein of the M13 family, which is specifically expressed in the early stages of the lymphoid progenitor, thus aiding in the identification of stages in B lymphocyte development (49). CD10 is widely used to distinguish most cases of ALL from other hematologic malignancies, and is commonly used in diagnosis via flow cytometry and monitoring of hematologic malignancies of B cell origin, in the categorization of the mature and blastic stage, and also for detection of measurable residual disease (50, 51). Originally identified in leukemic blasts, CD10 was later detected in cells from the prostate, kidney, intestine and endometrium (52, 53). The presence of CD10 in other cells suggests a varied role that is not specifically restricted to hematologic malignancies. Biologically, its main function is to metabolize polypeptides through peptide cleavage between hydrophobic residues, leading to the inactivation of a variety of physiologically active neuropeptides (54).

In the context of cancer, CD10 activity and its high expression has been correlated with a poor prognosis and decreased survival in a variety of malignancies, through mechanisms that include therapeutic drug and radiation resistance, increased tumor grade and a more aggressive phenotype (invasion and metastasis) (54–60). In the ontogeny of B lymphocytes, CD10, present in pre-B lymphocytes, is transiently expressed during different stages of maturation and disappears in mature B lymphocytes. In this sense, by evaluating the kinetics during induction therapy, it was possible to observe a clear decline in the EV-CD10^+^ levels in B-ALL PB and B-ALL BM on D35. In parallel, a decline in EV-CD19^+^ was observed in B-ALL PB; while, in B-ALL BM, a distinct behavior was observed, with a decrease on D15 followed by an increase on D35 (**Figure 3**). In a similar way, the EV signature during induction therapy demonstrated that, on D0, a greater proportion of B-ALL patients exhibited high production of EV-CD10^+^ and EV-CD19^+^, in contrast to on D35 (**Figure 5**). Collectively, these findings may be indicative of the elimination or meaningful decrease of leukemic blasts on D35, with subsequent production of mature B lymphocytes and EV-CD19^+^ (mature B lymphocyte-derived EVs) in the medullary compartment.

The signature analysis also demonstrated important changes in the other EV populations during induction therapy. Where on D0, B-ALL PB presented a higher proportion of high-producers of EVs, followed by a decline on D15 and D35, on the other hand, B-ALL BM presented a lower proportion of high-producers of EVs on D0, followed by an increase on D15 and D35 (**Figure 5**). Interestingly, the analysis of the integrative network of EVs also exhibited notable changes during treatment, but with a distinct behavior. On D0, B-ALL PB patients exhibited a network of EVs that was characterized by a limited number of interactions. However, on D35, a network more like that of the control group was observed. This network was characterized by an increase of connections among EV populations, with emphasis on EVs derived from leukocytes (CD45^+^, CD66^+^, CD14^+^ and CD3^+^). In parallel, B-ALL BM presented a profile similar to that of B-ALL PB, but with a greater number of interactions, which can be explained by the greater complexity of the medullary microenvironment (**Figure 6**). Similar behavior was observed in a previous study, where on D35, the B-ALL patients exhibited a network of cytokines characterized by an increase of multiple connections. This was composed of greater interactions among the mediators of the different response profiles, suggesting the recovery of pro-inflammatory response (61).

The most critical issue to be highlighted and evaluated in our data is whether the EVs originate specifically from leukemic blasts or from another cellular source, albeit on a smaller scale. This is of great importance because, if the former is true, then EV-CD10^+^ and EV-CD19^+^ can be qualified as very promising biomarkers of diagnosis and therapeutic response in ALL. Aiming to answer this question, the double-positivity of EVs for the CD10^+^ and CD19^+^ markers was evaluated. Incredibly, our results demonstrate that, just like EV-CD10^+^ and EV-CD19^+^, the double positive EVs (EV-CD10^+^CD19^+^) were elevated in B-ALL PB patients at diagnosis, with a 5-fold magnitude of change in relation to the CG (**Figures 8A–D**). However, the ROC curve analysis revealed an even better clinical performance (AUC = 0.984) in discriminating B-ALL patients from CG (**Figure 8E**), compared to isolated EV-CD10^+^ (AUC = 0.860) and EV-CD19^+^ (AUC = 0,844) (**Figure 7**), highlighting the potential of these vesicles as biomarkers.

Although these results appear promising, they still require further investigation. Such investigations involve a richer analysis of the protein cargo of EVs, as well as their impact on the leukemic microenvironment. Although scarce, studies in B-ALL have demonstrated that EVs derived from leukemic blasts are enriched in tetraspanins (CD9, CD61 and CD81), adhesion molecules (CD29 and CD1446), in addition to lineage-specific markers (CD10, CD19 and CD22) (17, 62). Furthermore, proteomic analyze revealed that A Disintegrin and Metalloproteinase 17 (ADAM17) and Autophagy Related Protein 3 (ATG3) molecules were highly expressed in EVs derived from plasma of B-ALL patients, being found enriched in the Notch and autophagy pathways, respectively. In addition, ROC curve analyzes revealed that ADAM17 and ATG3 showed high clinical performance (AUC = 0.989 and AUC = 0.956, respectively), reinforcing that EVs enriched by the proteins may represent valuable biomarkers in B-ALL (63).

Noteworthy, this study has limitations: i) Since it is a segment study, in many cases, the sample volume was insufficient to perform EV assays. This ended up leading to a reduction in the study population, which compromised the analysis of association with the clinical prognosis; ii) Another limitation was the non-application of other methods for evaluation of EVs, as nanoparticle tracking analysis (NTA), and transmission electron microscopy (TEM) or scanning electron Microscopy (SEM), which would provide more accurate data on the size range and diversity of EVs; iii) Finally, given the absence of an ultracentrifuge, the isolation protocol applied was not that recommended by the International Society for Extracellular Vesicles (ISEV) guidelines (64), which could result in a lower yield in the purification of EVs and imply the co-isolation of potential contaminants.

However, it is important to highlight that this is a proof-of-concept study. Additional studies will be carried out to fill the gaps and correct the limitations left by this study. In this sense, from a larger study population, we will seek to carry out a richer characterization, from a phenotypic and protein cargo point of view, aiming to explore the impact of EVs on the clinical prognosis of patients with B-ALL undergoing chemotherapy and remission.

## Conclusion

Our data demonstrated that: (i) The B-ALL patients exhibited a decrease in EV-CD41a^+^ on D0 that is followed by a progressive increase on D15 and D35, indicating recovery of thrombopoiesis; (ii) The B-ALL patients showed a marked production of EV-CD51/61^+^, indicating greater activation of ECs; (iii) In our cohort, CD10 and CD19 were the most expressed markers in the leukemic blasts; (iv) EV-CD10^+^ and EV-CD19^+^ showed predominance in the Megamix beads size range of 100-200 nm, configuring them as “small vesicles”; (v) The B-ALL patients exhibited dynamic EV kinetics and signatures during induction therapy, exhibiting distinct profiles on D0 and D35; (vi) The B-ALL patients showed a marked increase in the number of connections on D35, displaying a biological network that was more similar to that of the control group; (vii) EV-CD10^+^CD19^+^ (double-positives) were also increased and exhibited excellent clinical performance and general accuracy for discriminating the B-ALL patients from the CG, and are possibly associated with unfavorable outcomes.

Finally, our data indicate that EVs represent a potential field of investigation in ALL. Future studies should explore the cargos carried by EVs-CD10^+^CD19^+^, how it affects the leukemic microenvironment and, ultimately, its potential as a combined diagnostic and prognostic biomarker for B-ALL. If successful, leukemic EVs could become a valuable liquid biopsy tool, allowing the real-time monitoring of malignancy progression.

## Data Availability

The original contributions presented in the study are included in the article/**Supplementary Material**. Further inquiries can be directed to the corresponding author.

## Supplementary material

Supplementary Figure 1 Analysis of strategies for the phenotypic characterization of EVs using flow cytometry.

Supplementary Figure 2 Analysis of strategies for the phenotypic characterization of EVs using flow cytometry according to size range.

Supplementary Figure 3 Fold change and performance of the extracellular vesicles CD41a^+^ and CD51/61^+^ as diagnostic biomarkers of B-ALL. The fold changes **(A)** and significance of fold changes **(B)** were performed in the peripheral blood of the B-ALL patients at the diagnosis baseline as described in the Materials and Methods section. Receiver operating characteristic (ROC) curve analysis was carried out to assess the performance of EV-CD41a^+^ and EV-CD51/61^+^ levels as diagnostic biomarkers for B-ALL **(C)**. ROC curves were assembled to define the cut-off points and calculate the following performance indices: sensitivity (Se), specificity (Sp), likelihood ratio (LR), the best cut-off point, as well as the area under the curve (AUC) and p-value as indicators of global accuracy, as described in the Materials and Methods section.
