# Supplementary figures and images for: Exploring cell-derived extracellular vesicles in peripheral blood and bone marrow of B-cell acute lymphoblastic leukemia pediatric patients: proof-of-concept study
Source: Front Immunol. 2024 Aug 21;15:1421036. doi: 10.3389/fimmu.2024.1421036 (PMC11371606; doi:10.3389/fimmu.2024.1421036)

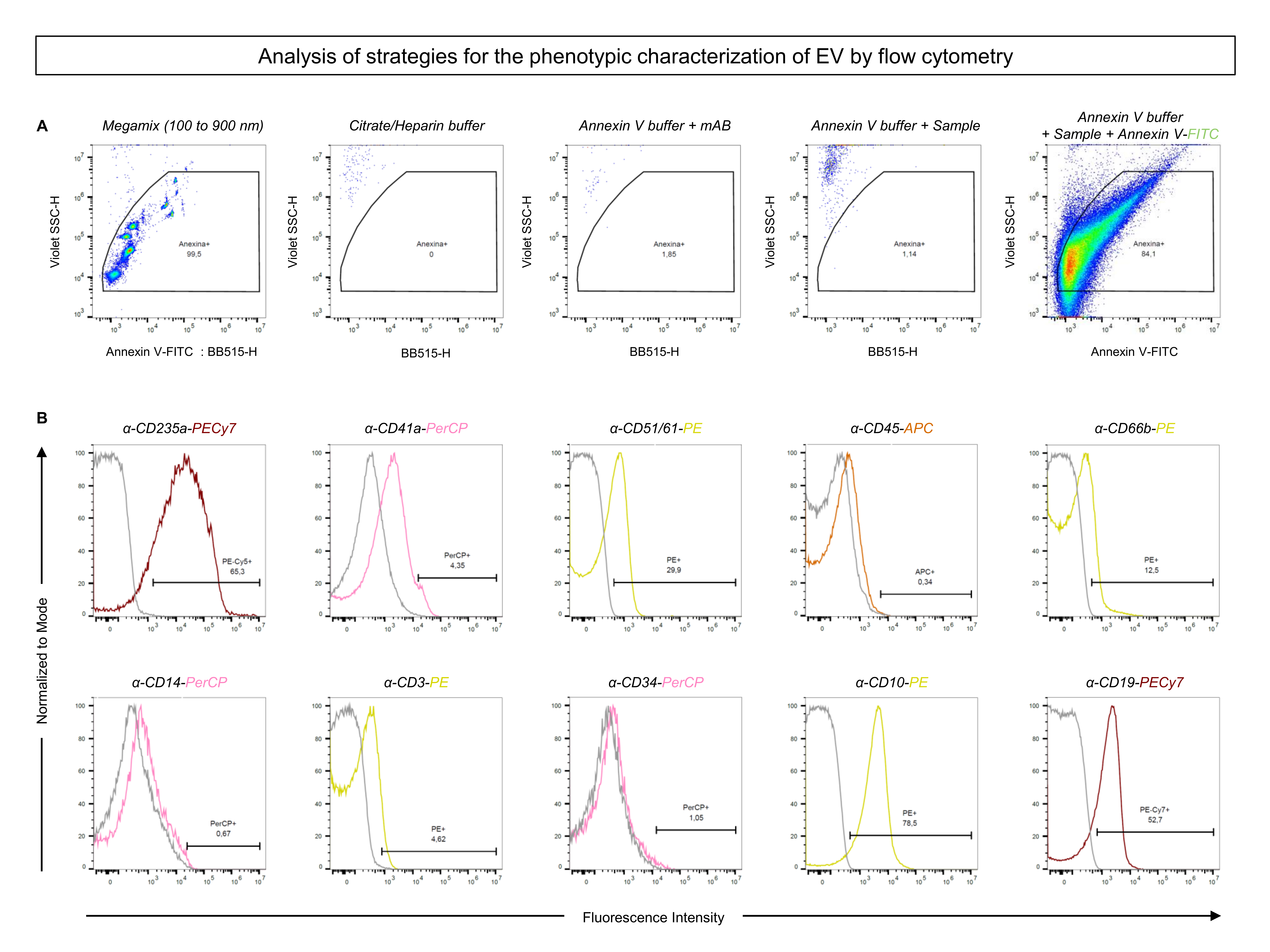

Supplement: Supplementary Figure 1 — Analysis of strategies for the phenotypic characterization of EVs using flow cytometry. [file Image1.tiff]

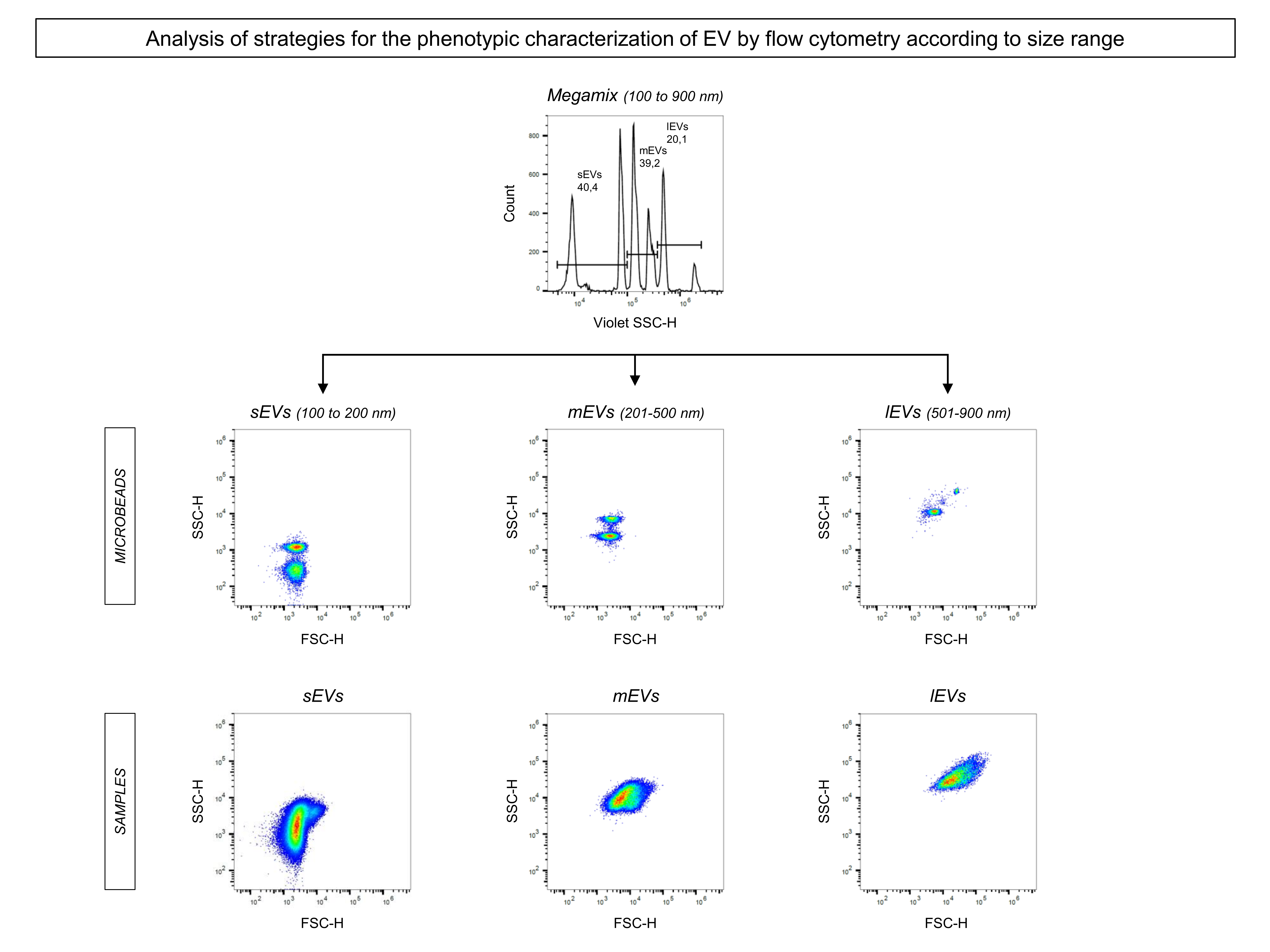

Supplement: Supplementary Figure 2 — Analysis of strategies for the phenotypic characterization of EVs using flow cytometry according to size range. [file Image2.tiff]

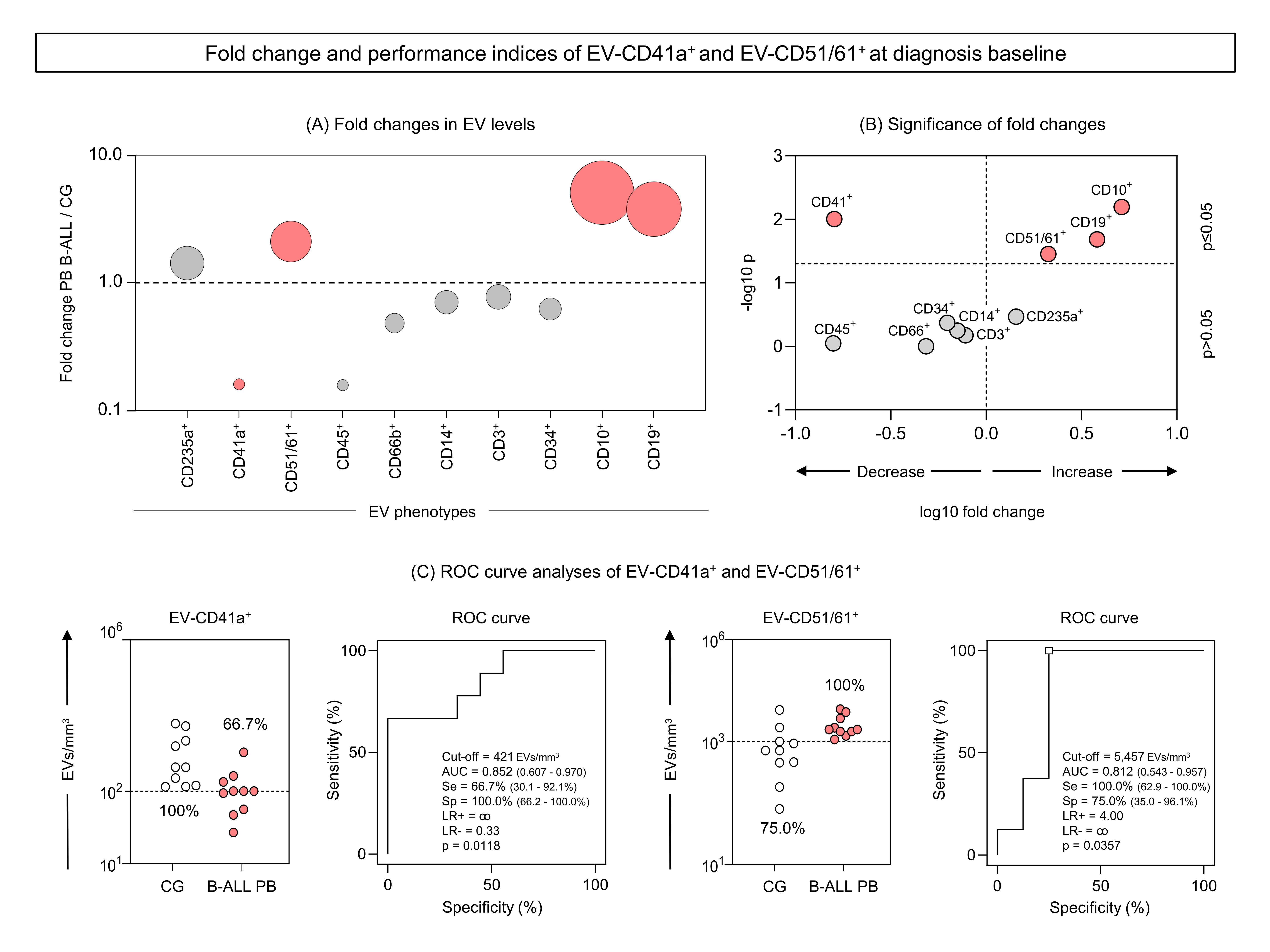

Supplement: Supplementary Figure 3 — Fold change and performance of the extracellular vesicles CD41a+ and CD51/61+ as diagnostic biomarkers of B-ALL. The fold changes (A) and significance of fold changes (B) were performed in the peripheral blood of the B-ALL patients at the diagnosis baseline as described in the Materials and Methods section. Receiver operating characteristic (ROC) curve analysis was carried out to assess the performance of EV-CD41a+ and EV-CD51/61+ levels as diagnostic biomarkers for B-ALL (C). ROC curves were assembled to define the cut-off points and calculate the following performance indices: sensitivity (Se), specificity (Sp), likelihood ratio (LR), the best cut-off point, as well as the area under the curve (AUC) and p-value as indicators of global accuracy, as described in the Materials and Methods section. [file Image3.tiff]
